# Process evaluation protocol plan for a home-based physical activity intervention versus educational intervention for persistent taxane-induced peripheral neuropathy (B-HAPI study): a randomized controlled trial

**DOI:** 10.1186/s12885-024-12444-x

**Published:** 2024-06-27

**Authors:** Samia Valeria Ozorio Dutra, Lauren Schwab, Jillian Coury, Ming Ji, Constance Visovsky

**Affiliations:** 1 Nancy Atmospera-Walch School of Nursing, University of Hawaii at Manoa, Honolulu, HI USA; 2 College of Nursing, University of South Florida, Tampa, FL USA; 3 Health Sciences, University of New Mexico, Albuquerque, NM USA

**Keywords:** Process evaluation, Taxane therapy, Home-based, Exercise, Intervention

## Abstract

**Background:**

Evaluation publications typically summarize the results of studies to demonstrate the effectiveness of an intervention, but little is shared concerning any changes implemented during the study. We present a process evaluation protocol of a home-based gait, balance, and resistance exercise intervention to ameliorate persistent taxane-induced neuropathy study according to 7 key elements of process evaluation.

**Methods:**

The process evaluation is conducted parallel to the longitudinal, randomized control clinical trial examining the effects of the home-based gait, balance, and resistance exercise program for women with persistent peripheral neuropathy following treatment with taxanes for breast cancer (IRB approval: Pro00040035). The flowcharts clarify how the intervention should be implemented in comparable settings, fidelity procedures help to ensure the participants are comfortable and identify their individual needs, and the process evaluation allows for the individual attention tailoring and focus of the research to avoid protocol deviation.

**Conclusions:**

The publication of the evaluation protocol plan adds transparency to the findings of clinical trials and favors process replication in future studies. The process evaluation enables the team to systematically register information and procedures applied during recruitment and factors that impact the implementation of the intervention, thereby allowing proactive approaches to prevent deviations from the protocol. When tracking an intervention continuously, positive or negative intervention effects are revealed early on in the study, giving valuable insight into inconsistent results. Furthermore, a process evaluation adds a participant-centered element to the research protocols, which allows a patient-centered approach to be applied to data collection.

**Trial registration:**

ClinicalTrials.gov NCT04621721, November 9, 2020, registered prospectively. Protocol version: April 27, 2020, v2.

**Supplementary Information:**

The online version contains supplementary material available at 10.1186/s12885-024-12444-x.

## Background

Breast cancer chemotherapy regimens vary, but many include taxane preparation [1]. Taxane-induced peripheral neuropathy is an important consequence of breast cancer therapy, leading to functional impairment and compromised quality of life. Chemotherapy-induced peripheral neuropathy (CIPN) occurs in up to 80–97% of patients with onset from week 1-101 with symptoms persisting until around 57 months [2, 3].

The “Home-based Physical Activity Intervention for Taxane-Induced CIPN” (B-HAPI) study is two-group, 16-week randomized clinical trial designed to address persistent taxane-induced peripheral neuropathy in women treated for invasive breast cancer. There have been only a limited number of original Randomized Controlled Trials conducted concerning this topic [4], particularly on proposing an exercise intervention specifically targeted towards persistent taxane-induced peripheral neuropathy using authenticated measures of gait and balance assessment.

Process evaluation is a systematic method for collecting, analyzing, and using data to examine the effectiveness of programs. Most evaluation publications report the results of studies to demonstrate the efficacy of an intervention. However, little is shared about protocol or other changes implemented during the research process that may influence the study outcomes. Often the mechanism of intervention delivery is overlooked as a critical aspect of evaluation, but instead should be treated as an important component of the overall intervention strategy, including the planning phase [5].

Implementing and obtaining process evaluation data helps to identify factors responsible for maintaining study integrity that may be implicated in determining the effectiveness of the intervention, the success or failure of an intervention, and for whom and under what circumstances the intervention is effective [6, 7].

In this paper, we present a process evaluation protocol of a home-based gait, balance and resistance exercise intervention to ameliorate persistent taxane-induced neuropathy study according to 7 key elements of process evaluation [6–8]. The 7 key process evaluation components that will determine intervention effectiveness are fidelity (quality), dose delivered (completeness), dose received on exposure and satisfaction, reach (participation rate), recruitment, and context.

## Methods

### Aim, design, and setting of the study

The process evaluation is conducted parallel to the longitudinal, randomized control clinical trial (B-HAPI study) whose objective is to examine the effects of the home-based gait, balance and resistance exercise program for women with persistent peripheral neuropathy following treatment with taxanes for breast cancer. The current process evaluation aims to: (1) monitor and assess the implementation of the home-based gait, balance, and resistance exercise program and (2) generate findings that aid in the interpretation and explanation of the program effects obtained in the parallel controlled trial. This model provides a conceptual framework for understanding the factors that affect the success or failure of a complex intervention. Data collection is structured using a triangulation design model [9]. The protocol had undergone previous scientific peer review as part of the grant application.

Process evaluation data are collected throughout the study as factors related to the successful completion of monthly questionnaires using Research Electronic Data Capture (REDCap), an electronic data capture tool hosted by University of South Florida. This data capture system maintains the standardized contact frequency of participants with the research team via telephone or videoconference, and health issues that can influence study-related processes. Results of the process evaluation are used to inform the intervention implementation and to perform midcourse corrections when fidelity of implementation is threatened (formative purposes). However, most process data will only be available following study intervention completion (summative purposes). Process data is ongoing and will be analyzed and interpreted prior to analysis of study outcomes. The hypothesis generated in the process evaluation derives from the adjustments in the implementation of the process only, and does not apply to not the original study hypothesis or results. These changes lead to new insights and hypotheses that can subsequently be statistically tested [5, 10].

#### Study design

**Fig. 1:**
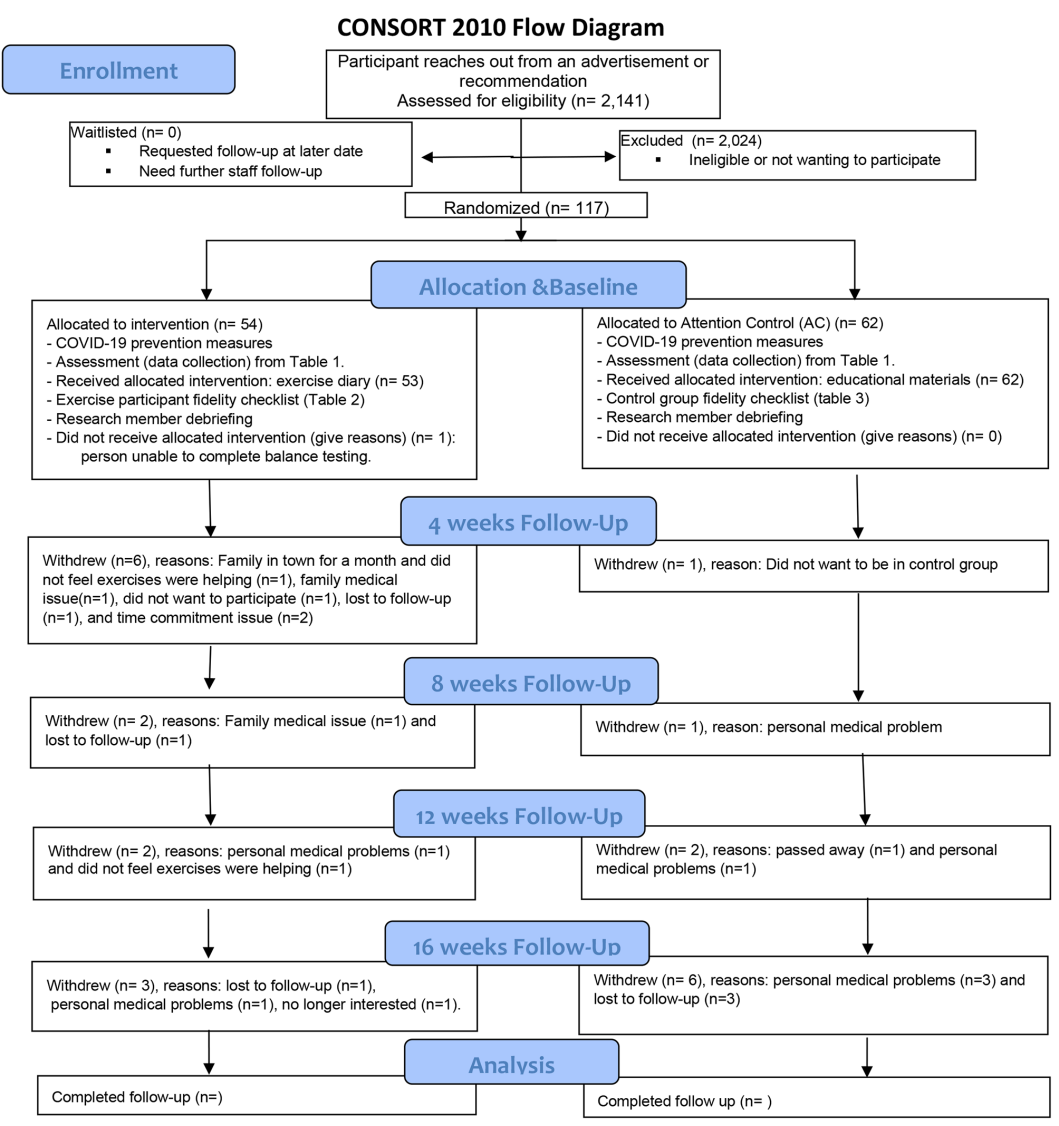
B-HAPI study CONSORT Flow Diagram. Displays the recruitment flow diagram for screening, randomized allocation per group, and follow up based on the Consolidated Standards of Reporting Trials (CONSORT).

A two-group longitudinal randomized controlled trial (RCT) was designed to address persistent chemotherapy induced peripheral neuropathy (CIPN) in women treated for invasive breast cancer with taxane-based chemotherapy. The B-HAPI study so far screened 1,889 people, including 94 people who are at least 6 months post-treatment and suffer from CIPN with a visual analog scale pain rating of ≥ 3. Figure 1 shows the CONSORT flow diagram of the study.

The study has the goal of recruiting 312 women in total, 156 in the intervention group and 156 in the attention control group. Power analyses determining the group sizes are described at the Statistical Analysis section. Breast cancer survivors are recruited from the regional community through breast cancer support groups, local institutions, social media campaigns, and recruitment flyers with the assistance of a local advertisement agency. Participants were randomized to either the intervention, consisting of a home-based exercise program, or an educational attention control group. Randomization to the study group was achieved using the REDCap randomization tool customized by the study statistician and REDCap specialist hosted at the University of South Florida [11, 12]. Protocol dictated that participants in both groups were to complete a total of five (5) appointments over the course of a 16-week period. Two in-person study appointments occurred once at the beginning and once at the end of the four (4) months. In between the two in-person appointments, participants in both groups had monthly phone calls scheduled at the 4-, 8-, 12-, and 15/16-week mark. The study finished recruiting and is in the last phases of the study with follow-up collection.

#### Setting

Following initial eligibility screening, the written informed consent, baseline data collection are conducted in person at the University of South Florida’s School of Physical Therapy and Rehabilitation Sciences Human Functional Performances Lab (HFPL) located on the university campus. The HFPL is a 6500 square foot research facility with a private space for consent and nerve conduction studies. It is equipped to assess performance, impairments, and functional limitations of neuromusculoskeletal conditions. Equipment in the HFPL that is utilized for this study includes: the BIODEX 3.0 computerized dynamometer to assess lower extremity muscle strength; the GAITRite System to assess gait; and the Neurocom Sensory Organization Test to assess balance. Nerve conduction studies are conducted at a private room in the HFPL by the collaborating study neurologist. Once baseline data are collected, group assignment (Exercise Intervention or Educational Attention Control) is revealed via RedCap. The data collector is blinded to study group assignment. Similarly, the 16-week (end of study) data collection is also performed in person with the same assessments as described above. All other data collection at 4, 8, and 12 weeks are done using a REDCap link sent to all study participants where the questionnaires can be accessed. Data is collected only in the United States. The Principal Investigator and statistician are blinded to the groups allocated intervention. Because this study has been evaluated as low risk by the university IRB, no unblinding guidelines were deemed necessary.

Participants randomized to the exercise intervention are instructed by the interventionist in all the exercises in the HFPL. The participant is given a tote bag with the B-HAPI research logo and the resistance bands and a paper exercise booklet for referral. Exercises are also recorded by the research team’s physical therapist on a YouTube channel and the link is provided to the participant. The exercise diary is provided to the is electronic through a RedCap link.

### Characteristics of the participants and measures

#### Population

Community-dwelling breast cancer survivors are recruited from the community. Female breast cancer survivors (≥ 21) who completed treatment for invasive breast cancer with taxane-based chemotherapy, and who have a peripheral neuropathy score of *≥* 3 by VAS rating were eligible for the study. Individuals with any disease (e.g. diabetes, HIV) that results in peripheral neuropathy or muscle weakness (chronic fatigue syndrome, multiple sclerosis, spinal cord tumors or injuries, stroke,); any disease that would preclude exercise (preexisting cardiopulmonary disease)) symptomatic lymphedema or at high risk for pathologic fracture are excluded. The study was approved by the University of South Florida Institutional Review Board (Pro00040035) and registered at ClinicalTrials.gov (Identifier: NCT04621721). If the study participants scored higher than 10 on the PHQ-9 or GAD-7 while answering the RedCap online forms, the Principal Investigator received an e-mail alert to inquire the reason for their high scores and make a decision about referral. Referrals to neurology, mental health professionals, and physical therapy were available through an affiliation with the University of South Florida healthcare network.

#### Attention control protocol

The attention control group participants received an educational intervention designed to equalize exposure to the exercise intervention protocol. Participants in this group received a journal binder in which to record their clinic and research appointments, pamphlets used for the educational attention control condition were from the American Cancer Society (ACS) and pertained to post-cancer care with additional supplemental information related to the ACS topics. Initially, the educational materials chosen consisted of (1) Nutrition: Eating Well After Treatment [13]; (2) Body Image and Sexuality After Breast Cancer [14]; (3) Life After Cancer/Follow-up Care [15]; and (4) Emotional and Social Issues After Cancer [16]. However, before the study was to commence, the SARS-CoV-2 pandemic struck the United States of America. As a result, the addition of COVID-19 Vaccinations: Myths vs. Facts and ‘Survivorship’ was added to the list of educational materials. In addition, participants were very interested in stress reduction techniques, so educational information on mindfulness-based stress reduction was also added. These topics were used as a substitute for those who chose to opt-out of any of the original topics.

The topics chosen were specially selected to provide relevant, timely information the individual can use in the cancer survivorship trajectory, while avoiding those related to exercise/physical activity to prevent contamination. Each control group participant received phone calls scheduled around data collection to equalize attention. Each phone call had a specific topic for that month and a trained member of the research team discussed the topic while providing additional insights in a semi-structured interview process. These educational sessions lasted approximately 20–35 min and occurred at the 4-, 8-, 12-, and 15-week mark. The attention control group members agreed to not begin a new exercise program or change their level of exercise during the study.

#### Exercise intervention protocol

The exercise intervention consists of a 16-week home-based exercise program meant to improve the participant’s gait, balance and lower extremity muscle strength. All material related to the exercise protocol was provided to the intervention group participants. The strength training exercises used progressive resistance flat bands for performing a variety of resistive exercises for the lower extremities, such as leg curls, lunges, and calf raises. The gait and balance exercises consisted of movements and postures that engaged varied sensory information by having participants perform static and dynamic tasks with eyes open/closed (visual), head steady or with head turns (vestibular), on firm surface/on foam (somatosensory). The exercise program contains detailed easy to follow demonstrations for each gait/balance training and resistance exercise training led by a physical therapist via a YouTube link. In addition, a pictorial exercise instruction booklet is also provided to participants for their reference. All exercise sessions are recorded in an Exercise Diary to provide a quantitative measure of exercise, as the prescribed exercises cannot be collected via any available device. Participants are instructed to complete the exercise diary for review at every data collection encounter. The intervention length is comparable with previous studies of exercise in persons with peripheral neuropathy [17–36] Intervention group participants are provided the resistance training bands of varying levels for the purpose of exercise progression, and wide, firm foam surface for the balance exercises. The intervention protocol begins with light warm-up and stretching activities followed by10 minutes each of gait/balance and 10 min of resistive (strength) training components. Telephone calls for follow-up to assist in surmounting barriers to exercise are conducted according to a standard schedule. The research team also offered video calls with participants to ensure proper exercise performance. The intervention nurse called each exercise participant one week after the baseline appointment to ensure exercise understanding and exercise diary completion. The study physical therapist also provided any needed consultations.

#### Data collection

Following informed consent, the following data is collected: age, gender, race, marital status, income level, employment status. Information concerning breast cancer stage, and hormonal status, type of breast cancer-related surgery, number of taxane cycles received, and current medications are also obtained.

**Fig. 2:**
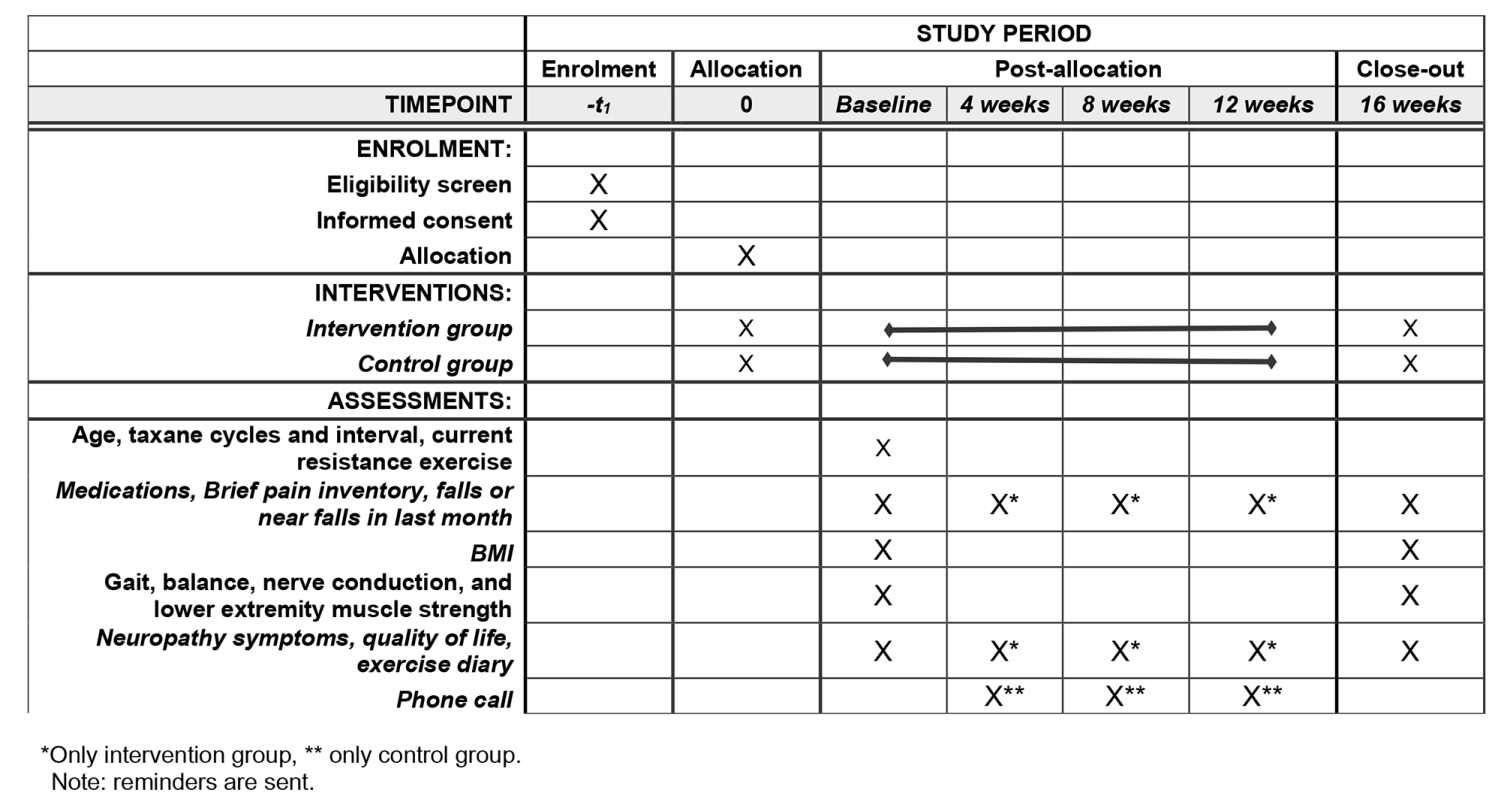
Standard Protocol Items recommended for Interventional Trials (SPIRIT) with the schedule of enrolment, interventions, and assessments. Displays Timeline for application of the standard protocol items. *Only intervention group, ** only control group. Note: reminders are sent

Assessments of lower extremity muscle strength [31], gait/balance [19, 26, 35], nerve conduction [20, 36], neuropathy symptoms [18], Brief Resilience Scale (BRS) [37], quality of life (QOL) [18], Generalized Anxiety Disorder (GAD-7) [38, 39], Patient Health Questionnaire (PHQ-9) [40, 41] are collected in person at baseline. At 4 weeks, 8 weeks, and 12 weeks, measures of neuropathy symptoms, anxiety, depression, resilience and QOL are collected online via RedCap at the end of the intervention (16 weeks) all in-person assessments are repeated as in the baseline measures. The assessments performed and instruments validity are described at Table 1 per time point. And Fig. 2 through the Standard Protocol Items recommended for Interventional Trials (SPIRIT) with the schedule of enrolment, interventions, and assessments.

**Table 1:** Instruments validity/reliability and time of measures

Variable	Instrument	Time of Measure *
Lower extremity muscle strength	*Isokinetic dynamometry* (Biodex 3.0) Hip flexors, hip abductors, knee flexors, knee extensors, and ankle dorsiflexors will be tested. A composite strength score for each lower extremity will be calculated for each extremity. *R & V ICC = 0.91–0.99* [31]	Baseline & 16 weeks
Gait and Balance	Gait analysis will be performed using a *GAITRite System* with 3D motion capture with integrated force platform. Gait variables to be used in analysis are ankle plantar/flexor torque & power [19, 26, 35] Sensory organization test (computerized dynamic posturography) for balance (*Neurocom Balance Master*, Clackamas, OR) [19]. The composite score comes from 6 conditions from eyes-open and eyes-closed derived from the sensory organization test through the computer algorithm that will be used as the balance variable in analysis. *ICCs were 0.62 (95% CI: 0.04, 0.80) for the eyes open and 0.80 (95% CI: 0.62, 0.90) for eyes closed tests.*	Baseline & 16 weeks
Nerve conduction	Nerve conduction studies of the sural & peroneal nerve action potentials will be tested at the USF Department of Neurology. Tests of nerve conduction have been successfully used to monitor change over time in studies of peripheral neuropathy from taxanes [20, 36].	Baseline and 16 weeks
Neuropathy Symptoms	*FACT-Taxane Additional Concerns* subscale^53^ Addresses symptoms specific to neuropathy. Likert scale: 0 (not at all) − 4 (very much). Symptom score can range from 0–4 with higher scores indicating more neuropathic symptoms. *r = 0.84–0.88, concurrent validity established* [18]	Baseline, 4, 8, 12, 16 weeks
Quality of Life	*FACT-Taxane* (version 4)^18^. A total Quality of Life score can be obtained by summing the subscale scores and will be used for in the data analysis. *r = 0.84–0.88, concurrent validity established.*	Baseline, 4, 8, 12, & 16 weeks
Exercise Diary	Intervention participants report frequency and perceived intensity	Baseline, 4, 8, 12, & 16 weeks
Anxiety	Generalized Anxiety Disorder 7-item (GAD-7) scale reflects on anxiety symptoms over the prior 2-week period. The cut-off scores of 5, 10, and 15 correspond to mild, moderate, and severe anxiety symptoms, respectively [38]. Reliability among cancer patients: Cronbach’s α = 0.88 [39].	Baseline, 4, 8, 12, & 16 weeks
Depression	Patient Health Questionnaire (PHQ-9) reflects on depression symptoms over the prior 2-week period [40]. A score over 10 indicated potential depression. Reliability among cancer patients: Cronbach’s α = 0.84 [41].	Baseline, 4, 8, 12, & 16 weeks
Resilience	Brief Resilience Scale (BRS) provides a total score of resilience. A score of 1.00-2.99 indicates low resilience, 3.00-4.30 indicates normal resilience, and 4.31-5.00 indicates high resilience. Reliability in various populations: Cronbach’s α ranging from 0.80 to 0.91 [37].	Baseline, 4, 8, 12, & 16 weeks
	**Control Variables**	
Age Taxane cycles and interval	Will be obtained by patient report Number of taxane cycles received and interval since last treatment will be collected.	Baseline Baseline
Medications Pain BMI Current resistance exercise Falls or near falls in last month	Medications used for neuropathy pain will be monitored and documented throughout the study, and coded into drug classifications, and dosage change/no change tracked for analysis. *Brief Pain Inventory* assesses severity of pain, impact of pain on daily function, location of pain, pain medications and amount of pain relief in the past 24 h. Cronbach alpha 0.77 to 0.91 [21]. A portable Tanita Body Composition Analyzer will be used to obtain each participant’s weight and BMI through bioelectrical impedance [23, 33]. Self–report (yes/no) Self-report (yes/no) [25, 34, 47]	Baseline, 4, 8, 12, & 16 weeks Baseline, 4, 8, 12, & 16 weeks Baseline & 16 weeks Baseline Baseline, 4, 8, 12, & 16 weeks

Individual semi-structured interviews by group assignment occurs on a regular basis at baseline, 4 weeks, 8 weeks, 12 weeks, and 16 weeks with all participants. The intervention group is asked about their ability to engage in the exercise program over the past few weeks, any barriers to exercise they have experienced, and strategies to overcome these barriers.

The attention control and intervention phone calls utilize standardized scripts and take a similar length of time at the same time intervals to equalize contact with both groups and avoid attention bias. The attention control script consists of the educational topics as noted above about barriers and strategies in the survivorship trajectory. The educational topics specifically avoid those related to exercise/physical activity to prevent contamination. Educational pamphlets of these topics are placed in the planners given to the attention control group. A review the assigned topic is provided during the scheduled attention control phone call, and the participant is engaged in a discussion of the topic and any questions are answered.

#### COVID-19 pandemic impact

While the overall COVID-19 pandemic has been resolved, it remains important to discuss the impact of the pandemic on the study processes. The study start was delayed for 4 months due to the 2020 acute COVID-19 outbreak which resulted in the closure of in-person university research activities. Once the study could begin recruitment, the research team took steps to mitigate COVID-19 infection transmission, as this occurred before vaccine approval. These steps included mask mandates for all research staff in contact with participants, the provision of clean, disposable masks for patients upon arrival, hand sanitization stations, procedures for sanitizing all surfaces and equipment before and after participant appointments, and the institution of a COVID-19 risk assessment questionnaire. For 2021 and 2022, those measures continued to be implemented until masks were not mandatory in our clinics, approximately mid-2022. However, aseptic techniques continued to be implemented as needed.

### Process description

#### Program implementations as planned

**Fig. 3:**
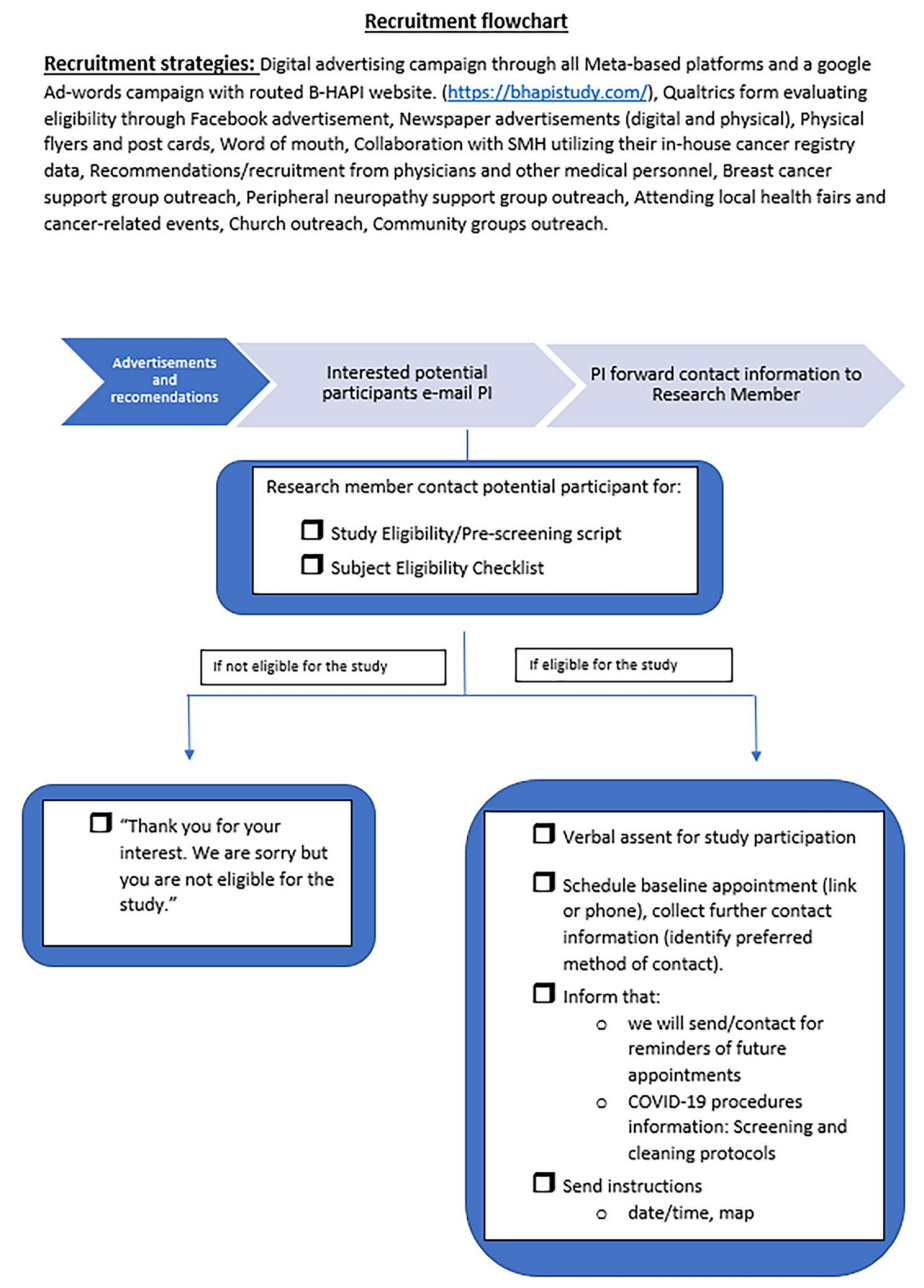
Recruitment. Reports detailed information and transcript for recruitment and enrollment in the study

A graphical presentation of the recruitment and data collection is provided as flowcharts (Figs. 2 and 3). The flowcharts clarify how the intervention should be implemented in comparable settings, revealing important aspects necessary to reach optimal performance and quick adjustments. Prior to starting recruitment, the research team assessed the fidelity of the intervention by use of a fidelity checklist developed by the PI. The fidelity checklist is utilized at regular weekly intervals throughout the study for training any new staff, for re-training and ensuring compliance with the intervention procedures.

**Fig. 4:**
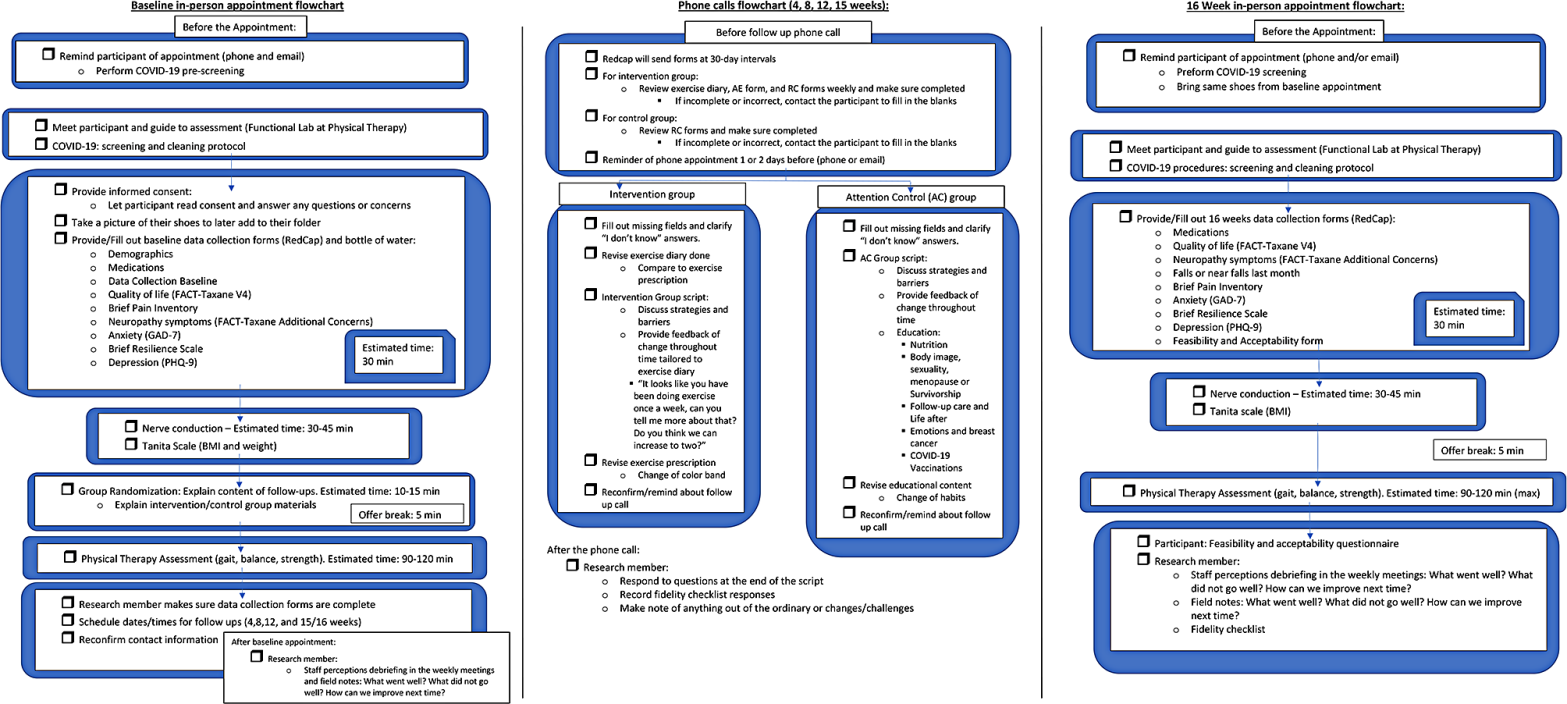
Baseline and follow-up flowcharts. Displays detailed information of the procedures during baseline and follow-up appointments. Both groups has the same baseline and final follow-up procedure (16 weeks), but differ in the follow-up for the 4,8,12, and 15 weeks

First, through social media marketing efforts, the participant reaches out the research team to obtain additional study information and to assess for interest and study eligibility. The team then explains the study objectives and requirements as well as triaging COVID-19 symptoms/risks during the active COVID-19 infection and quarantine period to ensure participants and team safety. Upon confirming eligibility (Fig. 3), the participants baseline lab visit is scheduled for data collection (Fig. 4).

The physical therapy lab team performing data collection, the study statistician and the primary investigator are blind to whether the participant is allocated to the intervention or control group at baseline and follow ups. Only the study research manager and research assistants are aware of the participants allocation as they proceed with the instructions and implementation of the exercise diary and educational materials for the attention group.

The participants provide data via a fidelity instrument (Tables 2 and 3, according to the designated group) and the research team members proceeded with debriefing. These procedures beyond the data collection helps to ensure the participants are comfortable and identify any of their individual needs, which helps building relationship rapport and avoid attrition rates.

**Table 2:** Fidelity instrument - intervention group

ID#:_____________			Date: __________________ Week #:___________
Did not cover	Covered partially	Covered fully	
			A. Introduction
1	2	3	Inquire about how the study is going for participants.
1	2	3	Answer any questions participant had regarding surveys or exercise diary.
			B. Stretching
1	2	3	Discuss about stretching prescription
1	2	3	Ask if participant feel any pain during stretching routine and, if yes, assisted in brainstorming ways to alleviate pain.
1	2	3	Remind participant to fill out their exercise diary.
			C. Gait and Balance (G&B)
1	2	3	Discuss barriers and strategies to overcome issues with performing G&B.
1	2	3	Ask if participant feel exertion during G&B exercises and adjust exercise prescription accordingly.
1	2	3	Ask if participant feel pain during G&B routine and assist in brainstorming ways to alleviate pain.
			D. Strength Training
1	2	3	Discuss barriers and strategies to overcome issues with performing strength exercises.
1	2	3	Ask if participant feel exertion during strength exercises and adjust exercise prescription accordingly.
1	2	3	Ask if participant feel pain during the strength routine and assist in brainstorming ways to alleviate pain.
			E. Additional Questions
1	2	3	Discuss any changes in participants health.
1	2	3	Discuss any additional questions or concerns by the participant.
1	2	3	Remind participant to fill out their exercise diary.
1	2	3	Remind participant to fill out the last two pages of the exercise diary if the participant has done any other exercises or if the participant had experienced any illnesses/injuries.
			E. Other Aspects of the Session
1	2	3	Registered comments about the session

**Table 3:** Fidelity instrument - attention control

ID#:________________			Date: ______________________ Week #:__________________
Did not cover	Covered partially	Covered fully	
			A. Introduction
1	2	3	Inquire about how the study is going for participants.
1	2	3	Answer any questions participant had regarding surveys
1	2	3	Ask whether the participant has read the educational material
1	2	3	Answer any questions the participant had regarding the educational material
			B. Educational Topic
1	2	3	Introduce the topic
1	2	3	Assess comfort level discussing the topic
			C. Summary of Topic
1	2	3	Give brief summary of topic along with asking questions imbedded within the summary
1	2	3	Ask about and discuss any concerns or barriers the participant experiences regarding the topic
1	2	3	Review strategies to overcome these barriers
			D. Additional Resources
1	2	3	Review additional helpful resources available in the area related to the topic
1	2	3	E. Post-Educational Session Questions
1	2	3	Discuss any changes in participants health.
1	2	3	Discuss any additional questions or concerns by the participant.
1	2	3	Remind participant to review the materials for the next educational session prior to the phone call
			E. Other Aspects of the Session
1	2	3	Registered comments about the session

The fidelity instrument is administered according to the designated group assignment. (Tables 2 and 3) This procedure allows structured data collection from participants in both the intervention and control groups concerning perception of the intervention or control conditions, with an opportunity for any comments about the session.

The team members debriefing was done initially at the end of the each follow up until the staff were comfortable with the procedures. Currently a debriefing concerning the fidelity measure is conducted bi-weekly at the research team meeting. The meeting time ensures reflection and alignment to study focus and procedures, providing an opportunity for feedback meetings. During those meetings, the primary investigator receives a status update on the research study as well as additional details regarding additional aspects of the research, such as logistics for collecting data and returning data to the research team. Team members were ready to correct the implementation of the intervention if needed to ensure fidelity to the intervention. They kept track of the discussion topics and changes for evaluation purposes. The study has not yet experienced any significant protocol deviations.

#### Process evaluation

Throughout the research process shown in the flowchart (Fig. 3), different elements of the process evaluation components are implemented and used to collect process data. The tools to collect process data are based on the nature of the process evaluation questions (Table 4), this includes how to acquire valid, reliable information efficiently and with the least burden on those involved. In Table 4, the tools/procedures for collecting data, data sources and process evaluation questions are indicated for each process evaluation component.

**Table 4:** Process Evaluation Model

Key Process Evaluation Components	Process Evaluation Topic	Process Evaluation Questions	Data Collection Tools
Intervention Fidelity (Quality of Implementation)	1. Implementation as planned	1. To what extent were all elements of the 16-week-delivered program implemented as planned?	1. Flow diagram checklists, team member debriefs, intervention script, attention control script
Dose delivered (Completeness)	2. Steps of the protocol followed by research team	2. To what extent did the research team follow all steps of the protocol (depicted at flow diagrams)?	2. Flow diagram checklists,
Dose received (Exposure)	3. Compliance of participants to follow-up actions	3. To what extent were participants compliant with follow-up actions formulated in the intervention (exercise) plan and attention control plan?	3. Exercise diary 4. Attention Control Script
Dose received (Satisfaction)	4. Satisfaction of participants 5. Benefit to participants	4. To what extent were participants satisfied with the follow up actions? 5. To what extent did participants benefited from follow-up actions of the intervention (exercise) plan and attention control plan?	4. Participant satisfaction survey 5. Participants satisfaction survey
Reach (Participation Rate)	6. Number of participants enrolled 7. Reasons non-participation 8. Completion steps protocol 9. Reasons drop-out	6. What proportion of the intended target population participated? 7. What were the reasons for non-participation? 8. What proportion of the participants people completed all steps of the intervention (exercise) plan and attention control plan? 9. What were the reasons for drop-out of participants enrolled?	6. Trial database, CONSORT flow diagram 7. CONSORT flow diagram, Intervention script and attention control script, research member debriefing 8. Intervention script and attention control script, adherence rates from exercise diary 9. CONSORT flow diagram, Intervention script and attention control script, research member debriefing
Recruitment	10. Recruitment procedures	10. What procedures were used to recruit female breast cancer survivors who completed taxane-based chemotherapy for participation?	10. Research Protocol
Context (General)	11. Implementation issues 12. Contamination	11. What barriers and facilitators influenced implementation of the intervention (exercise) plan and attention control plan? 12. To what extent did the control group receive the intervention or similar types of exercises (contamination)?	11. research team debriefing, Notes research team 12. research team debriefing, Notes research team

Quantitative data will be analyzed using the software package SPSS for windows computing descriptive statistics with means and frequencies, the attrition rate and follow-up contacts. We will compare both groups and test the efficacy of the 16-week delivered program of gait/balance training plus resistance exercise in increasing muscle strength, improving gai/balance and nerve induction parameters, decreasing neuropathy symptoms, increasing quality of life and resilience, and decreasing anxiety and depression while controlling for age, BMI, number of taxane cycles and intervals, neuropathic pain, neuropathy/pain medications, current resistance exercise participation and falls/near falls experienced.

The qualitative data collected by open-ended question in the fidelity checklist and teams notes throughout the process evaluation will be used for the individual attention tailoring and focus of the research to avoid protocol deviation. Content analysis on the notes about participants commons concerns will allow major themes to emerge from the data [42]. A narrative report will summarize the description of the procedures.

### Statistical analysis

Power analyses were performed through a Monte Carlo simulation approach with the software *Mplus* to calculate sample size [43, 44], incuding recommended variance of the population parameters. Observations were spaced at 0, 4, 8, 12, and 16, weeks with the number of weeks since baseline as the time metric to evaluate the efficacy of the 16-week intervention. To reflect an effective randomization of participants to conditions, we modeled no mean difference between treatment and control conditions at baseline, and the difference in slopes between the treatment and control conditions during the intervention period (γ_11_) is the focal parameter to be adequately powered. Given α = 0.05, a two-tailed hypothesis test, and the view that a power value of 0.80 will be adequate to detect a treatment effect, a minimum sample of *N* = 312 participants (based on recruitment of 2 or more participants per week for 3 years) with 20% attrition, 10% periodic non-response. A full-information maximum likelihood approach for an intent-to-treat analysis, a Monte Carlo simulation with 10,000 replications suggests we will be able to detect a minimum standardized effect of 0.30 with a probability of correctly rejecting a false null (power) of 0.81. If the recruitment rate is closer to 3 per week resulting in a sample of *N* = 468, the minimum detectable standardized effect is 0.25. By including additional control variables (all ES’s = 0.10), the minimum-detectable effect sizes decrease to 0.27 and 0.22, respectively. Topic relevant meta-analyses reported effect sizes for exercise intervention effects on similar outcomes to range between ES = 0.30 to ES = 0.0.84 [45]. The prospective power analysis suggests that our study is well positioned to detect effect sizes even at the lower end of this reported range.

In order to test the efficacy of the 16-week-delivered program of gait/balance training plus resistance exercise, we will use a intent-to-treat (ITT) analyses to evaluate the effect of the intervention using the Exercise Diary for change in outcomes at post-intervention and at follow-up and a structural equation modeling (SEM) to explore the covariates of the intervention effect. The aforementioned analyses provide a generalized mixed model that allows to modeling both time-varying covariates (e.g., pain, medications, BMI, falls) and individually varying covariates (e.g., age, taxane cycles, years since treatment completion, baseline resistance exercise); adjust for loss of power and bias derived from attrition and periodic non-response; utilize a non-normal link function from non-normally-distributed outcomes; and, consider individual differences in baseline outcomes and improved outcomes from the intervention by allowing initial status and change over time to be random (latent) variables. The intention-to-treat analyses are based on differential improvement outcomes between the treatment and control conditions during the 16-week intervention efficacy period.

We will also evaluate for differences in muscle strength, gait/balance, sensory (sural) and motor (peroneal) nerve conduction, peripheral neuropathy symptoms, quality of life (QOL), resilience (BRS), anxiety (GAD-7), and depression (PHQ-9) between groups (exercise-intervention vc educational-intervention, control group) while controlling for age, Body Mass Index, taxane cycles and intervals, neuropathic pain, neuropathy/pain medications, current resistance exercise participation and falls/near falls experienced.

Additional parameters are included to evaluate the time-varying controls (pain, medication use, BMI, fall) and time-invariant controls (age, taxane interval/cycles, baseline resistance exercise). Control for these potential covariate effects reduces potential bias to the slope parameters central to the test of study aims and increases statistical power.

A certified research associate and statistician are dedicated to the role of data management. The process evaluation is periodically analyzed through descriptive statistics analysis (quantitative data) and content analysis (qualitative data). The process evaluation analysis allows individual attention while focusing on research to avoid protocol deviation. This study has been evaluated as low risk by the university IRB and no stopping guidelines to terminate the trial were deemed necessary.

## Discussion

This paper describes the process evaluation protocol plan for the B-HAPI study: Home-based physical activity intervention for taxane-induced CPIN: A randomized controlled trial (RCT). Beyond focusing on publishing the outcomes, publishing the process flow diagram and evaluation model favors replication of a complex longitudinal clinical trial study. This allows midcourse correction when fidelity of the implementation is threatened with data analysis and interpretation before the outcomes of the effect of the study. Considering that most summative process data is not processed or available until after completion of the proposed intervention [6], the process evaluation is critical for the success and replication of the study.

The incorporation of process evaluation elements in the process supports the implementation of the intervention key components. After all, it ensures that quantitative and qualitative data supports an understanding and assurance of the quality and process of the implementation are gathered [46].

The process evaluation allows the team to systematically register information and procedures applied during the recruitment process and factors influencing the intervention implementation, which allows a proactive approach to avoid protocol deviations. This allows a seamless documentation of midcourse correction, non-participation and drop-outs during recruitment, intervention, and follow-up.

By following the flow diagram consciously incorporating the process evaluation key components, the team gathered valuable information. Whenever there were conflicting opinions regarding adjustments of the process, the research team revisited the study hypothesis/objective. The research financial institution and IRB should be consulted for any potential significant adjustment.

Regarding the breast cancer chemotherapy regimens, taxanes are known to induce peripheral neuropathy toxicity leading to lower extremity muscle weakness, impaired balance, pain, numbness, and decreased vibration or touch sensation [47–49]. Currently, there is no evidence-based preventative or treatment strategies available [50, 51] and a limitation of current publications is the lack of a clear theoretical framework in the development process [52]. Studies in this field may benefit from a thorough process evaluation publication to determine factors that facilitate or hinder the intervention.

Lastly, by tracking the implementation of an intervention continuously, favorable, or unfavorable intervention effects can be clarified early on in the study, which leads to valuable insights into contradictory results. The use of a mixed methods approach provides a key strength to the process evaluation by providing an understanding of the processes and experiences of participants with both interventions. As a general principle, combining quantitative and qualitative methods increases validity more so than utilizing either one alone [46].

In conclusion, the publication of the process evaluation plan adds transparency to the findings of clinical trials and favors process replication in future studies. The authors believe every study and intervention management follows a structured protocol procedure, barriers, and adjustments as part of the studies ethics and procedures. However, adding transparency by publishing the process implemented and not only the outcomes validity and reliability is a practice that still needs to be instilled in the research community.

A process evaluation has many uses depending on the main objective, the available resources, the type of intervention, and where it will be implemented. It also adds a participant-centered component into the research, bringing the patient-centered model into data collection. While executing the process evaluation, one challenge is to consider whether interim adjustments and changes can be made to ensure that the exercise and educational intervention will be implemented with fidelity without jeopardizing the study protocol’s integrity. The team ensured fidelity through consultation with the study physical therapist co-investigators, statistician and study neurologist prior to any significant adjustments. In addition, physical therapists not part of the study team were used to assess features of the exercise protocol for the intervention group and suggest and necessary adjustments.

For dissemination, the team plans to publish the data in publications and presentations in several venues, including national and international professional meetings. For the patients, we communicate with them routinely through the newsletter, which is published periodically every month, and will publish a final newsletter in December 2024.

### Limitations

A limitation is the execution of the process evaluation by the research team, which may introduce bias. However, acknowledging this possibility and introducing consultation to experts on the decision-making process of adjustments (a peer review by an independent researcher component) helps to reduce this risk.

Randomized clinical trials are only designed to test interventions with a positive effect, making generalization of results difficult because the study population differs greatly from the population treated in normal life. Additionally, trials are not usually able to answer the questions practitioners, decision-makers, or consumers ask. For an insight into long-term outcomes and endurance of the outcomes at 16 weeks, follow up should extend beyond 16 weeks.

### Electronic supplementary material

Below is the link to the electronic supplementary material.

Supplementary Material 1

Supplementary Material 2

## Data Availability

The data are available from the authors upon reasonable request.

## Abbreviations

RCT: Randomized Clinical Trial
B-HAPI: Home-Based Physical Activity Intervention
HFPL: Human Functional Performances Lab
QOL: Quality of Life
BRS: Brief Resilience Scale
GAD-7: Generalized Anxiety Disorder 7-item scale
PHQ-9: Patient Health Questionnaire
COVID-19: Coronavirus Disease 2019
CONSORT: Consolidated Standards of Reporting Trials
BMI: Body Mass Index
