# Supplementary material for: Process evaluation protocol plan for a home-based physical activity intervention versus educational intervention for persistent taxane-induced peripheral neuropathy (B-HAPI study): a randomized controlled trial
Source: BMC Cancer. 2024 Jun 27;24:777. doi: 10.1186/s12885-024-12444-x (PMC11212161; doi:10.1186/s12885-024-12444-x)
Supplement: Supplementary file 2 — Supplementary Material 2 [file 12885_2024_12444_MOESM2_ESM.pdf]

UNIVERSITY OF SOUTH FLORIDA

Informed Consent to Participate in Research and Authorization to Collect, Use, and Share your Health Information

Information to Consider Before Taking Part in this Research Study

Title: Home-Based Physical Activity Intervention (B-HAPI) for Persistent Taxane-Induced Neuropathy

Pro # 00040035

Overview: You are being asked to take part in a research study. The information in this document should help you to decide if you would like to participate. The sections in this Overview provide the basic information about the study. More detailed information is provided in the remainder of the document.

Study Staff: This study is being led by Constance Visovsky who is a professor/researcher at the College of Nursing. This person is called the Principal Investigator. Other approved research staff may act on behalf of the Principal Investigator.

Study Details: This study is being conducted at University of South Florida and is supported/sponsored by the National Institutes of Health. The purpose of the study is to test the effects of a home-based exercise program as compared to an educational group. Participants in the exercise group will be given exercise training, resistance training bands and a firm foam surface. Participants in the educational group will receive educational materials related to cancer. The length of your participation in the study is 16 weeks.

Participants: You are being asked to take part because you were treated with taxane-based chemotherapy for your breast cancer and you have peripheral neuropathy. We want to find out if the study exercise program helps people who have peripheral neuropathy.

Voluntary Participation: Your participation is voluntary. You do not have to participate and may stop your participation at any time. There will be no penalties or loss of benefits or opportunities if you do not participate or decide to stop once you start.

Benefits, Compensation, and Risk: We do not know if you will receive any benefit from your participation. There is no cost to participate. You will be compensated $25 for each study visit for a total of $125 ($25 x 5). If you withdraw for any reason from the study, you will be compensated $25 for each study visit you complete. This research is considered minimal risk. Minimal risk means that study risks are the same as the risks you face in daily life

Confidentiality: Even if we publish the findings from this study, we will keep your study information private and confidential. Anyone with the authority to look at your records must keep them confidential.

Study Procedures: What will happen during this study?

You will be assigned to one of the following groups: B-HAPI Exercise Group or Educational Attention

Group.

If you are in the B-HAPI Exercise Group,

- You will receive an exercise program through a link or by DVD. You will also receive a resistance band and a wide, firm foam surface. The exercise program includes gait/balance training and resistance exercises to follow 5-7 days per week. The exercise program includes easy to follow demonstrations of the exercises led by a physical therapist. Each session begins with a light warm-up and stretching activity, followed by 25 minutes gait and balance exercises, and 25 minutes of strength training.
- You will be asked to record your exercise at all sessions in your Exercise Diary.
- There will be a total of 5 study visits (Baseline, 4, 8, 12 and 16 weeks) with the baseline and 16-week visit lasting 2.5 – 3 hours and the 4, 8, and 12-week appointments lasting 45-60 minutes each. The baseline and 16-week study visits will take place at the University of South Florida (USF) School of Physical Therapy and the 4, 8, and 12-week appointments will be telephone meetings. At each study visit, you will be asked to complete questionnaires about your pain, quality of life, and medication changes/fall risk update.
- At the Baseline and 16 weeks study visits, your muscle strength, gait, balance, and nerves will be tested at the School of Physical Therapy.

If you are in the Educational Attention Group, you will be asked to not change your previous exercise or physical activity routine while you are in this study.

- There will be a total of 5 study visits (baseline, 4, 8, 12 and 16 weeks) with the baseline and 16 week visit lasting 2.5 – 3 hours and the 4, 8, and 12 week appointments lasting 45-60 minutes each. The baseline and 16-week study visits will be at the University of South Florida School of Physical Therapy and the 4, 8, and 12-week appointments will be telephone meetings.
- You will receive a journal to record your clinic appointments.
- At each visit, you will also receive an American Cancer Society pamphlet to be placed in your journal and complete questionnaires. The research assistant will discuss the information in each American Cancer Society pamphlet. The questionnaires will ask about your pain, medication changes, fall update and quality of life.
- At the Baseline and 16 weeks study visits, your muscle strength, gait, balance, and nerves will be tested.
- You will also receive telephone follow-up calls one week following data collection, bi-monthly.

Total Number of Participants

About 312 individuals will take part in this study at USF.

Alternatives / Voluntary Participation / Withdrawal

You do not have to participate in this research study.

You should only take part in this study if you want to volunteer. You should not feel that there is any pressure to take part in the study. You are free to participate in this research or withdraw at any time. There will be no penalty or loss of benefits you are entitled to receive if you stop taking part in this study.

You can decide after signing this informed consent document that you no longer want to take part in this study for any reason at any time. If you decide you want to stop taking part in the study, tell the study staff as soon as you can.

- We will tell you how to stop safely.
- If you decide to stop, you can continue getting care from your regular doctor.

Please note, even if you want to stay in the study, there may be reasons we will need to withdraw you from the study. You may be taken out of this study if we find out it is not safe for you to stay in the study or if you are not coming for the study visits when scheduled. We will let you know the reason for withdrawing you from this study.

Benefits

We are unsure if you will receive any benefits by taking part in this research study.

Risks or Discomfort

Breach of confidentiality is a potential risk. Study materials will have unique study numbers and will kept secured in locked files.

If you are part of the exercise group, the following risks may occur:

- Falls are a potential risk if you have balance difficulties or become dizzy. To minimize the risk, you should have a sturdy chair nearby and a person with you while you are exercising.
- Sore muscles from the exercises.

Compensation

You will be compensated $25 for each study visit for a total of $125 ($25 x 5). If you withdraw for any reason from the study, you will be compensated $25 for each study visit you complete.

Costs

It will not cost you anything to take part in the study.

Privacy and Confidentiality

We will do our best to keep your records private and confidential. We cannot guarantee absolute confidentiality. Your personal information may be disclosed if required by law. Certain people may need to see your study records. These individuals include:

- The research team, including the Principal Investigator, study coordinator, research nurses, and all other research staff.
- Certain government and university people who need to know more about the study. For example, individuals who provide oversight on this study may need to look at your records. This is done to make sure that we are doing the study in the right way. They also need to make sure that we are protecting your rights and your safety.
- Any agency of the federal, state, or local government that regulates this research. This includes the Department of Health and Human Services (DHHS) and the Office for Human Research Protection (OHRP).
- The USF Institutional Review Board (IRB) and its related staff who have oversight responsibilities for this study, and staff in USF Research Integrity and Compliance.

Your identifiers might be removed from your private records. Your information could be used and/or distributed to another investigator for future research studies without additional consent from you or your Legally Authorized Representative.

We may publish what we learn from this study. If we do, we will not include your name. We will not publish anything that would let people know who you are.

What if new information becomes available about the study?

During the course of this study, we may find more information that could be important to you. This includes information that, once learned, might cause you to change your mind about being in this study. We will notify you as soon as possible if such information becomes available.

You can get the answers to your questions, concerns, or complaints.

If you have any questions, concerns or complaints about this study, call Dr. Connie Visovsky at 813-974-3831. If you have questions about your rights, complaints, or issues as a person taking part in this study, call the USF IRB at (813) 974-5638 or contact by email at RSCH-IRB@usf.edu.

Authorization to Use and Disclose Protected Health Information (HIPAA Language)

The federal privacy regulations of the Health Insurance Portability & Accountability Act (HIPAA) protect your identifiable health information. By signing this form, you are permitting the University of South Florida to use your health information for research purposes. You are also allowing us to share your health information with individuals or organizations other than USF who are also involved in the research and listed below.

In addition, the following groups of people may also be able to see your health information and may use that information to conduct this research:

- The medical staff that takes care of you and those who are part of this research study.
- The USF Institutional Review Board (IRB) their related staff who have oversight responsibilities for this study, including staff in USF Research Integrity and the USF Health Office of Clinical Research.
- The Data Safety Monitoring Board.
- The National Cancer Institute, sponsor of the study.
- Each research site for this study including USF.
- Any laboratories, pharmacies, or others who are part of the approved plan for this study.

Anyone listed above may use consultants in this research study, and may share your information with them. If you have questions about who they are, you should ask the study team. Individuals who receive your health information for this research study may not be required by the HIPAA Privacy Rule to protect it and may share your information with others without your permission. They can only do so

if permitted by law. If your information is shared, it may no longer be protected by the HIPAA Privacy Rule.

By signing this form, you are giving your permission to use and/or share your health information as described in this document. As part of this research, USF may collect, use, and share the following information:

- Your research record

You can refuse to sign this form. If you do not sign this form you will not be able to take part in this research study. However, your care outside of this study and benefits will not change. Your authorization to use your health information will not expire unless you revoke (withdraw) it in writing. You can revoke your authorization at any time by sending a letter clearly stating that you wish to withdraw your authorization to use your health information in the research. If you revoke your permission:

- You will no longer be a participant in this research study;
- We will stop collecting new information about you;
- We will use the information collected prior to the revocation of your authorization. This information may already have been used or shared with others, or we may need it to complete and protect the validity of the research; and
- Staff may need to follow-up with you if there is a medical reason to do so.

To revoke your authorization, please write to:

Principal Investigator
For IRB Study # Pro00040035
University of South Florida
College of Nursing
12901 Bruce B Downs Blvd, MDC 22
Tampa, FL 33612

While we are conducting the research study, we cannot let you see or copy the research information we have about you. After the research is completed, you have a right to see the information about you, as allowed by USF policies.

Consent to Take Part in Research and Authorization for the Collection, Use and Disclosure of Health Information

I freely give my consent to take part in this study. I understand that by signing this form I am agreeing to take part in research. I have received a copy of this form to take with me.

____ ____________

Signature of Person Taking Part in Study/Authorization Date

____

Printed Name of Person Taking Part in Study/Authorization

Statement of Person Obtaining Informed Consent and Research Authorization

I have carefully explained to the person taking part in the study what he or she can expect from their participation. I confirm that this research participant speaks the language that was used to explain this research and is receiving an informed consent form in their primary language. This research participant has provided legally effective informed consent.

____ ____________

Signature of Person Obtaining Informed Consent/Authorization Date

____

Printed Name of Person Obtaining Informed Consent/Authorization
